# Alteration of DSS-mediated immune cell redistribution in murine colitis by oral colostral immunoglobulin

**DOI:** 10.1186/1471-2172-14-10

**Published:** 2013-02-20

**Authors:** Peggy Bodammer, Elisabeth Zirzow, Sebastian Klammt, Claudia Maletzki, Claus Kerkhoff

**Affiliations:** 1 Division of Gastroenterology, Department of Medicine II, University of Rostock, Rostock 18057, Germany; 2 Department of Immunology, Project group “Extracorporal Immunomodulation”, Fraunhofer Institute for Cell Therapy and Immunology, Rostock 18057, Germany; 3 Centre for Coordination of Clinical studies, University of Rostock, Rostock, 18057, Germany; 4 Department of General, Vascular, Thoracic and Transplantation Surgery, Section of Molecular Oncology and Immunotherapy, University of Rostock, Rostock, 18057, Germany

**Keywords:** DSS-colitis, Recovery, Colostrum, Secretory immunoglobulin A, Bovine lactoferrin, *γδ*- T-cells, Myeloid-derived suppressor cells, Outbred NMRI mice

## Abstract

**Background:**

Oral bovine colostrum prophylaxis accelerates the recovery of dextran sulfate sodium (DSS)-induced colitis. In the present study the beneficial effects on acute intestinal inflammation of two major colostral components, secretory immunoglobulin A and lactoferrin, were investigated. Outbred NMRI mice received whole bovine colostrum (BC, 20 mg/kg body weight), colostral bovine lactoferrin (bLf, 150 mg/kg), or secretory immunoglobulin A (sIgA, 1–2 mg/kg body weight) daily by oral gavage, either two weeks before induction of colitis (prophylaxis) or after disease establishment (therapy). Bovine serum albumin (BSA, 150 mg/kg body weight) and immunoglobulin G (IgG, 1 and 2 mg/kg body weight) served as protein controls. Colitis was induced by providing 5% DSS solution *ad libitum* for seven days.

**Results:**

Compared to BSA, BC therapy improved occult blood, stool consistency, and clinical recovery from colitis but did not prevent initial weight loss. In contrast, administration of bLf did not influence the course of colitis in either the prophylactic or the therapeutic setting. Therapeutic application of sIgA promoted weight gain in the recovery phase of colitis but failed to improve other clinical parameters. Prophylactically-fed sIgA influenced immune cell redistribution, normalized peripheral blood CD11c^+^CD83^+^ mature dendritic cells, modulated colonic immune cell infiltration, and altered the numbers of both DSS-induced regulatory *γδ* TCR^+^ T cells and CD11b^+^Gr-1^+^ myeloid suppressor cells in the lymph nodes and spleens of mice.

**Conclusions:**

These data demonstrated the potential of colostrum in disease recovery and epithelial homeostasis following intestinal injury. Colostral sIgA failed to improve acute disease activity but promoted weight gain and modulated immune cell responses that are involved in the genesis of colitis.

## Background

Crohn’s disease and ulcerative colitis are chronic inflammatory disorders of the gut that cause major life-long disability. Afflicting mostly young people at an age when they are most active both in their private and professional life, inflammatory bowel disease (IBD) represents an important public health problem affecting the patients education, working abilities, social life and quality of life. The cause of IBD is multiple and so far not completely understood. However, genetic factors, environmental factors and the gut bacteria play a role in disease development.

Conventional therapy of active IBD pre-dominantly target anti-inflammatory immune responses, largely due to cytokine release within the intestine. Thus, therapeutic treatment mainly includes anti-inflammatory drugs, immunosuppressants, biologic agents, and antibiotics [1]. However, these agents may cause severe adverse effects and are therefore not suitable for long-term treatment of IBD. Moreover, conventional drugs block manifestation or consequences of inflammation in acute disease. Thus, there is a necessity for therapeutic strategies that target improvement of impaired barrier function in remission. Suitable candidates are dietary supplements and food components such as colostrum. Although natural therapies are commonly associated with lower toxicity and fewer side effects than conventional drugs, the scientific proof of their effectiveness and safety is demanded [2].

Colostrum, the secretion produced by the mammary glands during the first three days post-partum, contains many functional nutrients. These include immunoglobulins, growth factors, and antimicrobial peptides. The potential of colostrum to affect gastrointestinal infections and to reduce the incidence of immune-mediated diseases is well established [3,4]. In accordance with this, we recently demonstrated the protective effect of orally applied colostrum in a murine colitis model [5]. Beneficial effects were characterized by improvement of clinical colorectal inflammation symptoms and by the induction of immunoregulatory mechanisms, predominantly of the innate immune system arm. Thus, identification of factors responsible for preventing experimental colitis might provide the basis for developing a long-term treatment regimen.

In IBD patients, local production of polymeric IgA (pIgA) is altered and this affects both immunological homeostasis and humoral immune responses [6,7]. IgA does not activate the classical complement pathway and inhibits the production of pro-inflammatory cytokines in response to lipopolysaccharide (LPS), thereby maintaining mucosal integrity [8]. SIgA preparations from colostrum are active against various microbial antigens and inhibit adhesion and invasion by enteropathogenic *Escherichia coli in vitro*[9,10]. Colostral sIgA influences the development of the gastrointestinal immune system in milk-fed infants; however, little is known about the effectiveness of oral sIgA in maintaining gut barrier functions in adults.

Another colostral peptide with a broad range of immunomodulatory properties is lactoferrin (Lf), a member of the iron-binding glycoprotein family. Lf is predominately found in mucosal secretions and neutrophilic granules [11]. Lf concentration and iron saturation differ among species; bovine (bLf) and human (hLf) Lfs show strong sequence homology [12]. BLf has been reported to stimulate mucosal and systemic immune responses when given orally [13,14]. Moreover, there is experimental evidence that Lf reduces the severity of colitis in rodents [15,16].

In the present study, we explored the potential of orally applied colostral bLf and sIgA for modulating immune responses and recovery from dextran sodium sulfate (DSS)-induced murine colitis. Whole bovine colostrum (BC) improved the clinical severity of colitis, whereas bLf had no effect. SIgA influenced DSS-mediated immune cell redistribution and specifically altered colonic cell infiltration. Therefore, it might be an interesting candidate for promoting regeneration from acute colitis.

## Results

### Therapeutic application of colostrum and sIgA improved clinical recovery from colitis

**Figure 1:**
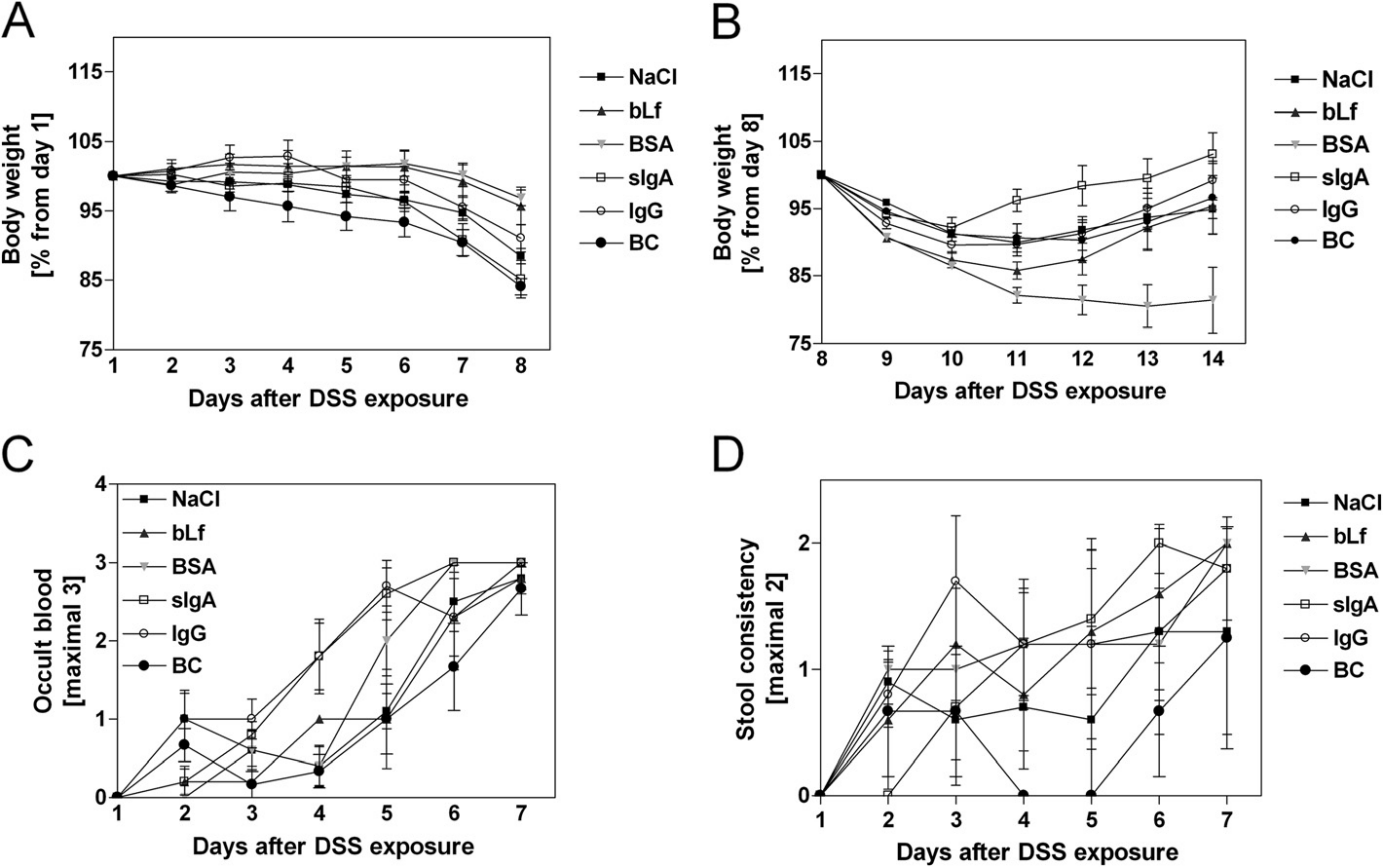
**Influence of therapeutically applied colostral compounds on clinical severity of DSS-induced colitis. **The indicated therapeutics (bLf, sIgA, IgG, and BC) and controls (NaCl and BSA) were applied parallel to DSS treatment. Weight loss was either represented during the induction phase of colitis as percentage of body weight from day 1 (**A**) or during the recovery phase as percentage from day 8 (**B**). Clinical disease activity was determined by scoring changes in body weight, occult blood (**C**) and stool consistency (**D**) as shown in Additional file 3. Values are means ± SD of n = 8 (NaCl), n = 6 (BC, sIgA, IgG), and n = 5 (bLf, BSA) mice.

After a single seven-day treatment of 5% DSS in drinking water, the mice showed signs of acute colitis including weight loss, bloody stools, and diarrhea. During the induction phase of colitis, neither the treatment (BC, sIgA, bLf) nor the control (NaCl, IgG, BSA) groups differed significantly with respect to body weight (Figure 1A). However, disease activity in the acute phase of inflammation (day 4 to day 7) declined only after BC-feeding owing to reduced occult blood (P = 0.008 *vs.* BSA, Figure 1C) and enhanced stool consistency (P = 0.028 *vs.* BSA, Figure 1D).

During the recovery phase, mice receiving therapeutic sIgA gained more weight than controls receiving NaCl (P = 0.077) or BSA (P = 0.002). BC and bLf showed only marginal differences from the control groups (NaCl, IgG, BSA). Since BSA-fed mice showed the most dramatic weight loss, up to 20% at day 14, the BC- and sIgA-induced recovery was not simply attributable to a feeding effect (Figure 1B).

### Therapeutic application of colostrum or colostral components had no effect on histopathological severity of colitis

In contrast to the clinical benefit, BC and sIgA did not reduce colon shortening at day 15 after DSS challenge. In all experimental groups, colon lengths were significantly lower than those in controls not treated with DSS (see Additional file 1). Histological examination of the colon at day 15 revealed no significant differences in total histological score or in grade and extent of inflammation (see Additional file 1).

### Therapeutic application of sIgA affected immune cell redistribution

**Figure 2:**
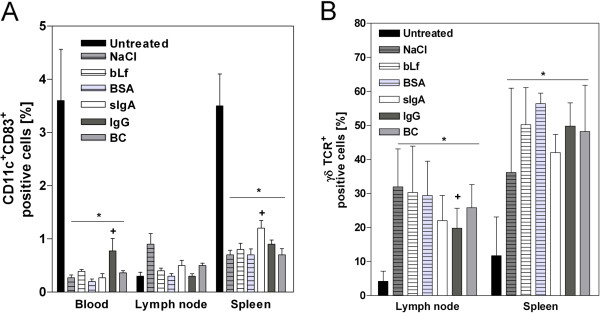
**Leukocyte distribution in peripheral blood, lymph nodes and spleen at day 15 after starting DSS exposure. **The indicated therapeutics (bLf, sIgA, IgG, and BC) and controls (NaCl and BSA) were applied parallel to DSS treatment. The untreated group represents healthy animals without any intervention. Values are presented as percent positive CD11c^+^CD83^+^(**A**) or *γδ*TCR^+^(**B**) of 10,000 measured events and given as mean ± SD; n = 12 (Untreated) n = 8 (NaCl), n = 6 (BC, sIgA, IgG), and n = 5 (bLf, BSA). *p < 0.05 *vs.* Untreated; +p < 0.05 *vs. *NaCl, Mann Whitney *U*-test.

The induction of colitis is characterized by a reduced number of mature CD11c^+^CD83^+^ dendritic cells (DCs) in the periphery and spleen and by a raised level in the mesenteric lymph nodes. Therapeutic application of Igs normalized the distribution of CD11c^+^CD83^+^ cells compared to NaCl controls by increasing their numbers in the spleens of sIgA-fed and in the peripheral blood of IgG-fed mice. Inflammation-induced elevation of DCs in mesenteric lymph nodes was exclusively found in NaCl controls; their numbers were not altered in the therapeutic groups (Figure 2A). In addition to DCs, *γδ* TCR^+^ T cells are significantly influenced by DSS-induced colitis [5], and the levels of this cell population were higher in the mesenteric lymph nodes and spleens of DSS-exposed mice than in untreated controls. However, except in the case of IgG, therapeutic application of BC or Lf did not significantly alter the DSS-mediated cell redistribution (Figure 2B). Taking these findings together, the observed clinical benefit of sIgA treatment could partly be explained by systemic immunological alterations.

### Effect of prophylactically applied secretory IgA on clinical severity of colitis

**Figure 3:**
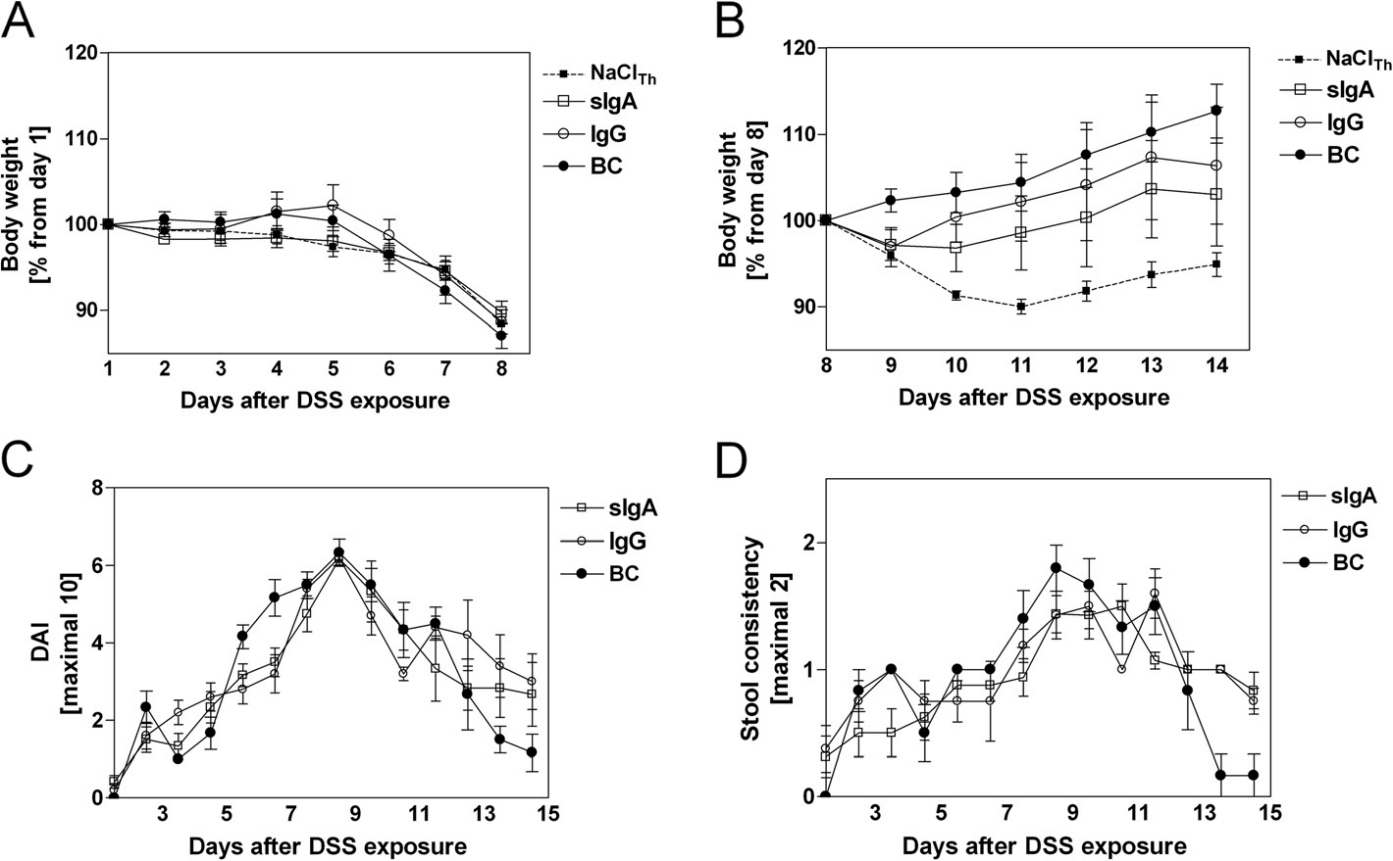
**Influence of prophylactically applied immunoglobulins and whole BC on clinical severity of DSS-induced colitis. **The indicated therapeutics (sIgA, IgG, and BC) and control (NaCl) were applied parallel to DSS treatment. Negative control was represented from the therapeutic setting (NaCl_Th_). Weight loss was either shown during the induction phase of colitis as percentage of body weight from day 1 (**A**) or as weight loss during the recovery phase as percentage body weight from day 8 (**B**). Clinical disease activity (DAI) (**C**) was determined by scoring changes in body weight, occult blood and stool consistency (**D**) as described in the Methods section. Values are mean ± SD of n = 8 (NaCl_Th_), n = 6 (BC), n = 6-8 (sIgA), and n = 5-8 (IgG) mice.

Next, we analyzed the potential of sIgA in a prophylactic setting. IgG served as control and BC as positive control. During the feeding period, sIgA and IgG were well tolerated by all mice and no side effects such as lethargy, abdominal pain or diarrhea were observed. Initial weight loss from day 1 to day 7 remained unchanged in all experimental groups (Figure 3A). In the recovery phase, weight gain was enhanced within the BC group until the end of the experiments (Figure 3B), and BC ameliorated clinical DAI (P = 0.025 *vs.* IgG) owing to improved stool consistency (P = 0.045 *vs.* IgG) (Figure 3C and D). Clinical recovery did not differ significantly between sIgA- and IgG-fed mice (Figure 3B and C).

### Prophylactic application of secretory IgA failed to improve histopathological changes of colitis but affected colonic cell infiltration

**Figure 4:**
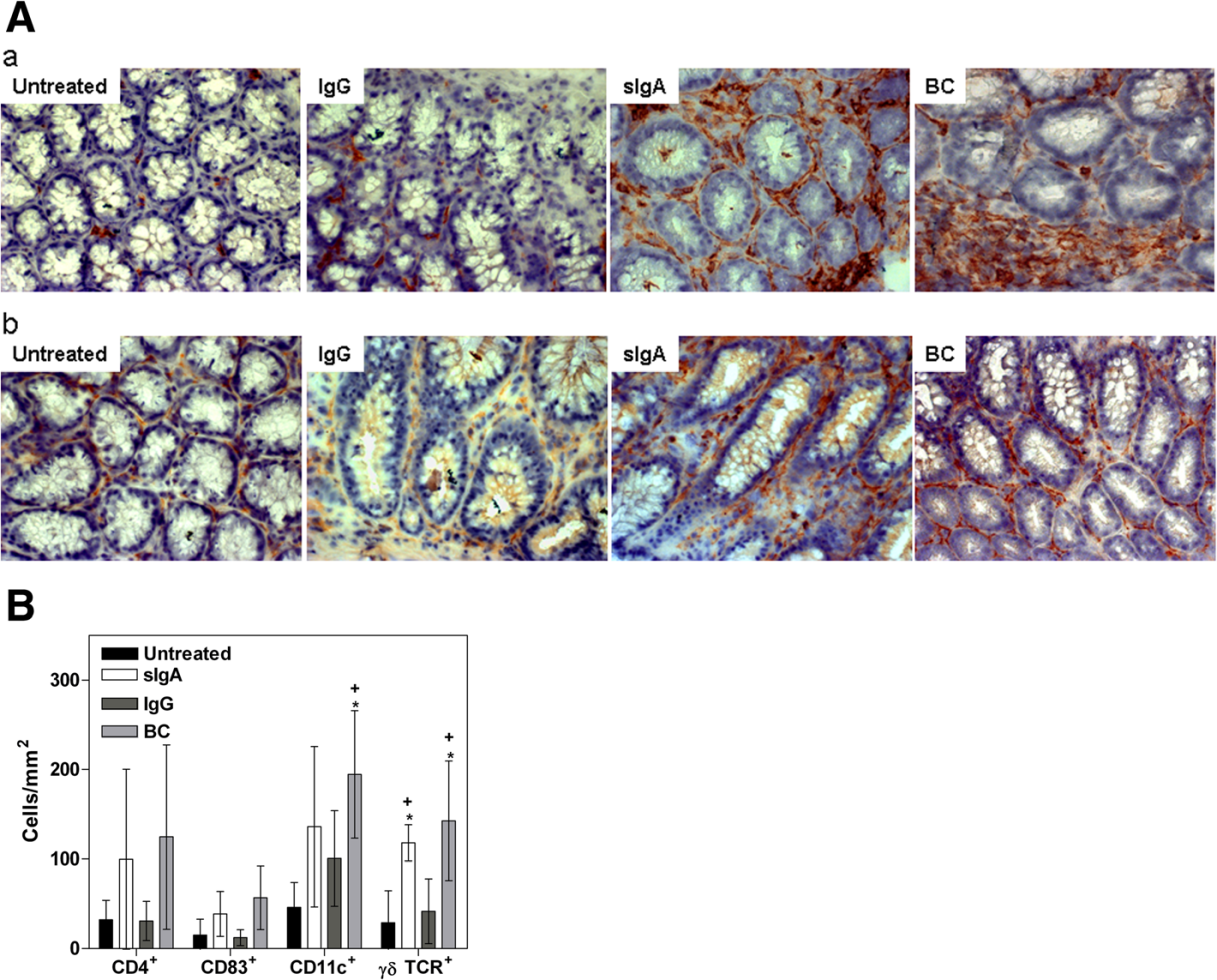
**Colonic infiltration of prophylactically-fed, DSS-exposed mice and untreated control without intervention at day 15. **The indicated therapeutics (sIgA, IgG, and BC) were applied parallel to DSS treatment. The untreated group represents healthy animals without any intervention. **A**: Immunohistochemical staining of **a**) CD11c^+^ mature dendritic cells or **b**) γδTCR^+^ T-cells in the colon of DSS-exposed mice compared to untreated, healthy controls. Representative images are shown in 200-fold magnification. **B**: Impact of prophylactic sIgA on cell distribution in lymph nodes and spleen at day 15. The indicated therapeutics (sIgA, IgG, and BC) were applied parallel to DSS treatment. The untreated group represents healthy animals without any intervention. Values are given as percent CD3^+^CD4^+^(A), γδTCR^+^(B), or CD11c^+^CD83^+^(C) positive cells of 10,000 measured events and given as mean ± SD. n = 6-8 (Untreated), n = 6 (BC, sIgA), and n = 5 (IgG) mice. *P < 0.05 *vs.* untreated, Mann Whitney *U*-test.Values are means ± SD of n = 6 (Untreated, BC, IgA) and n = 5 (IgG) mice.* *P* < 0.01 *vs.* untreated, ^+^*P* < 0.05 *vs.* IgG, Mann Whitney *U*-test.

Colon length (data not shown) and total median histological score did not differ significantly among any of the DSS-exposed experimental and untreated control groups (see Additional file 2). Therefore, a detailed immunohistochemical analysis from colonic sections was performed to unravel the observed clinical benefit (Figure 4A). The numbers of CD4^+^ and *γδ* TCR^+^ T cells and of CD11c^+^ and CD83^+^ mature dendritic cells were markedly higher in the BC and sIgA groups than in IgG-fed mice (Figure 4B). These data argued for a specific local immune modulation by BC and colostral sIgA.

### Prophylactically applied secretory IgA normalized DSS-mediated redistribution of immune cells

**Figure 5:**
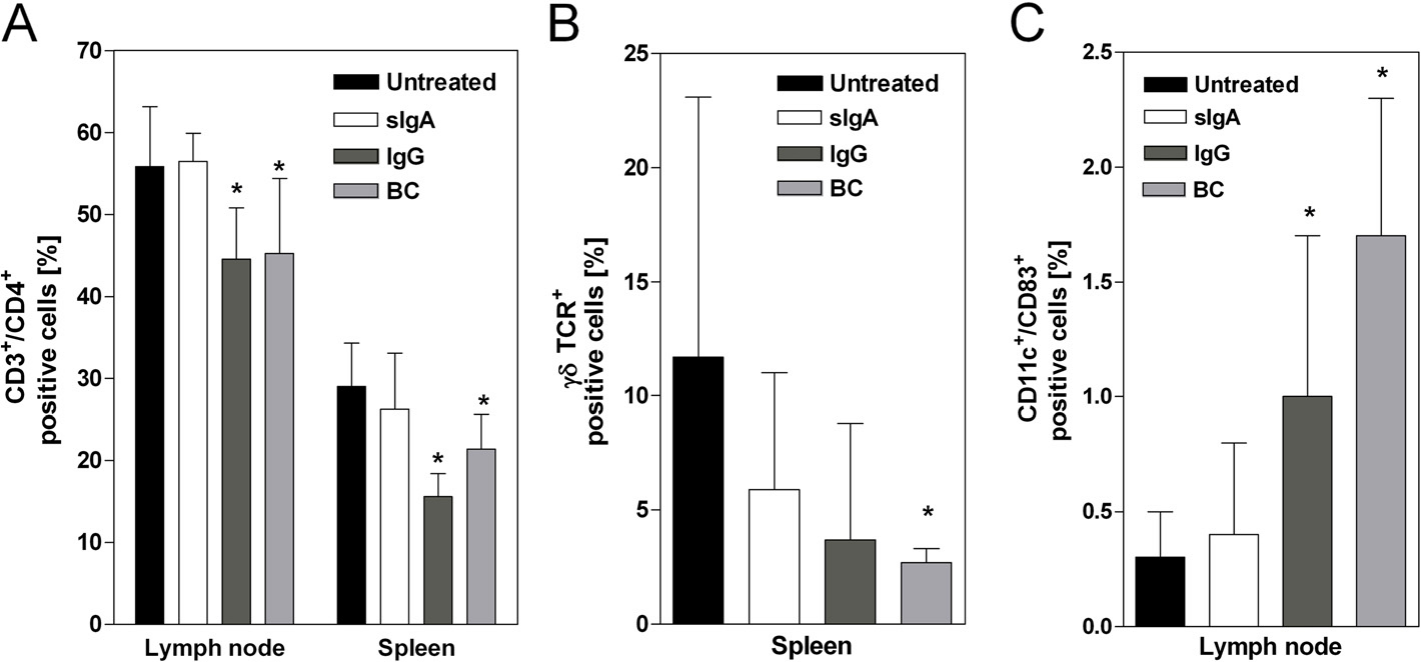
**Impact of prophylactic sIgA on cell distribution in lymph nodes and spleen at day 15. **The indicated therapeutics (sIgA, IgG, and BC) were applied parallel to DSS treatment. The untreated group represents healthy animals without any intervention. Values are given as percent CD3^+^CD4^+^ (**A**), γδTCR^+^ (**B**), or CD11c^+^CD83^+^ (**C**) positive cells of 10,000 measured events and given as mean ± SD. n=6-8 (Untreated), n=6 (BC, sIgA), and n=5 (IgG) mice. *P<0.05 *vs. *untreated, Mann Whitney *U*-test.

We next examined whether immunological effector cells were influenced by sIgA in other lymphatic organs. Prophylactic application of sIgA normalized T cell numbers in spleen and mesenteric lymph nodes. Levels of CD3^+^CD4^+^ cells were similar to those in the untreated control mice. Interestingly, neither IgG nor BC affected T cell numbers, indicating a specific sIgA-mediated response (Figure 5A).

Comparable though less pronounced effects were observed for splenic *γδ* TCR^+^ cells (Figure 5B). In mesenteric lymph nodes, a similar modulation of immune cell distribution was found. Here again, levels of mature CD11c^+^CD83^+^ dendritic cells were specifically normalized by sIgA prophylaxis (Figure 5C). Taken together, these data clearly pointed to immunological alterations by sIgA prophylaxis that influenced colonic inflammation.

### Prophylactically applied secretory IgA increased levels of DSS-induced myeloid-derived suppressor cells

**Figure 6:**
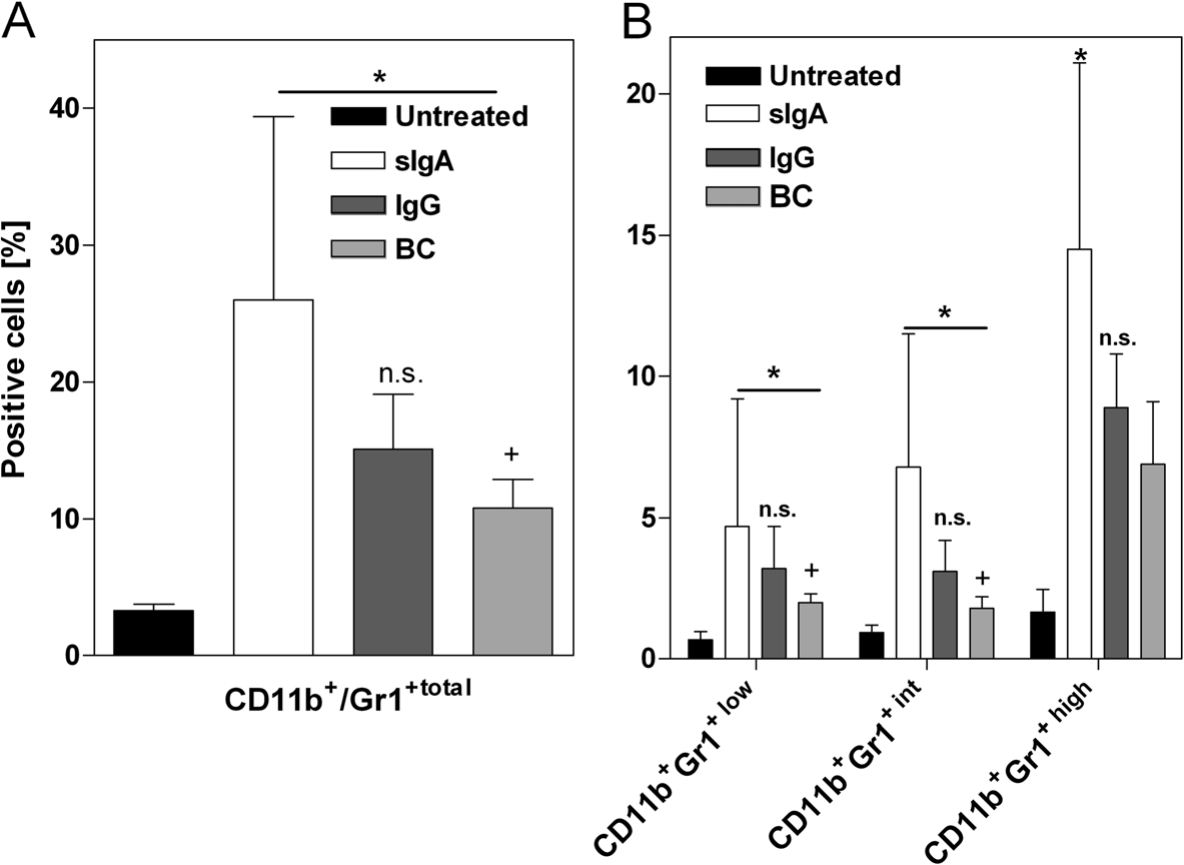
**Impact of prophylactic sIgA on DSS-induced myeloid-derived suppressor cells at day 15. **The indicated therapeutics (sIgA, IgG, and BC) were applied parallel to DSS treatment. The untreated group represents healthy animals without any intervention. Values are provided as percent total CD11b^+^Gr-1^+^ cells positive cells (**A**) or subpopulations with low, intermediate (int) or high CD11b^+^Gr-1^+^ co-expression in 10,000 measured events (**B**). Values are given as mean ± SD. n = 6-8 (Untreated), n = 6 (BC, sIgA), and n = 5 (IgG) mice. *P < 0.05 *vs. *untreated, ^+^*P* < 0.05 *vs.* sIgA, n.s. = not significant IgG *vs.* sIgA, Mann Whitney *U*-test.

Myeloid-derived suppressor cells (MDSC) represent another cell population that is altered during the course of colitis [5]. The MDSC population is heterogeneous and is characterized by CD11b^+^ and Gr1^+^ co-expression. These cells are induced by DSS exposure and accelerate recovery from colitis following transplantation [17]. Accordingly, the total levels of splenic CD11b^+^Gr1^+^ cells were higher in DSS-exposed mice than in healthy untreated controls on day 15 (Figure 6). While BC prophylaxis nearly normalized splenic CD11b^+^Gr1^+^ cell numbers, sIgA did not. In sIgA-fed mice, the numbers of MDSCs were massively increased (Figure 6A). It is notable that these effects were evident in all three subpopulations (CD11b^+^Gr1^+low, int, high^); MDSC levels were more than three-fold higher than in IgG and BC-exposed mice (Figure 6B).

## Discussion

As shown in a previous study, oral BC prophylaxis accelerates recovery from DSS-induced colitis [5]. However, most treatment regimens for IBD patients are not available before the onset of disease. Therefore, the present study was undertaken to evaluate the potential of whole BC and selected colostral compounds such as sIgA and bLf in a therapeutic setting. Both compounds display immunomodulatory potential [6,11], are resistant against proteolytic degradation, and accumulate in blood and intestinal tissue of mice [18,19].

The major finding of the study was that whole BC and in part colostral sIgA improved clinical recovery from colitis when applied therapeutically. This conclusion was confirmed by the enhanced weight gain of sIgA-fed mice and the lower disease activity (occult blood, diarrhea) in the BC-fed treatment group. Although BC and sIgA prevented neither the initial weight loss after induction of colitis nor the histological outcome of colitis, sIgA- and BC-fed mice recovered more quickly than control animals. In striking contrast, bLf failed to improve the clinical and histological course of colitis after both therapeutic and prophylactic applications (data not shown). This was surprising since bLf showed anti-inflammatory potential in DSS-induced colitis and stimulated mucosal and systemic immune responses when given orally [13,14]. In addition, reduced LPS-induced epithelial damage and barrier destruction have been reported [20], as well as decreased rectal bleeding and an improved histological score [15]. These findings, seemingly contradictory to our study, could be attributed to the fact that in these studies, human Lf with 7% iron saturation level was applied perorally twice a day. In contrast, we used Lf derived from bovine colostrum, and the level of iron saturation of bovine Lf was in range of 5 to 30%. Therefore, the discrepancy might be due to the therapeutic regimen rather than to the iron saturation level. Thus, increasing the number of Lf injections per day might have beneficial effects on the course of colitis. However, this was beyond the scope of the present study.

Another remarkable finding of our study was the effect of immunoglobulins on those immunological effector cells that most probably promote disease recovery. Mature DCs (CD11c^+^CD83^+^) and immunoregulatory *γδ* TCR^+^ T cells were almost normalized in lymph nodes and spleens in the sIgA and IgG groups. Similar data were obtained in the prophylactic setting. SIgA almost normalized the DSS-mediated cell redistribution (CD4^+^ T helper cells, *γδ* TCR^+^ T cells, CD11c^+^CD83^+^ mature dendritic cells) in lymph nodes and spleen.

Although *γδ* T cells are important in intestinal inflammation, the functional role of this cell population remains unknown. Under physiological conditions, they represent a minor subset within lymph nodes and spleens; however, they are induced in mice and men by inflammatory responses [21,22]. Peripheral human *γδ* T cells exert regulatory functions and suppress T helper cell proliferation [23]. Thus, the specific effects of sIgA on immune cell distribution can be regarded as favorable immune modulation of DSS-induced inflammation. In addition, colostral sIgA but not IgG influenced colonic cell infiltration. Just as whole BC is effective in preventing DSS-induced colitis, so sIgA treatment was characterized by massive increases in colonic CD11c^+^ and CD83^+^ DCs as well as *γδ* TCR^+^ T cells. Thus, the increase of local regulatory cells after BC prophylaxis was most likely to have been due to colostral sIgA rather than IgG. Activated *γδ* T cells have been shown to promote epithelial cell growth via the production of keratinocyte growth factor [24] and to regulate intestinal homeostasis [25], while depletion of *γδ* T cells worsened inflammation in mouse models of colitis [26,27]. Therefore, in accordance with these studies, the enhanced recovery from colitis was most probably mediated by immune cell modulation through colostrum and in particular colostral sIgA.

In our previous study, we hypothesized that MDSCs contributed to the clinical benefit of prophylactically applied colostrum [5]. Here again, sIgA prophylaxis specifically increased the number of splenic CD11b^+^Gr-1^+^ MDSCs. Although the function of CD11b^+^Gr-1^+^ MDSC in intestinal inflammation remains unclear, MDSCs accumulated in the bone marrow and spleen of DSS-exposed mice, and intravenous transplantation of DSS-induced splenic CD11b^+^Gr-1^+^ cells in C57Bl/6 mice resulted in advanced mucosal healing [17]. We therefore conclude that the strong induction of CD11b^+^Gr-1^+^ cells by sIgA contributed to the enhanced recovery from weight loss at later time points.

## Conclusions

In conclusion, we tested the immunomodulatory potential of sIgA and bLf for improving the severity of DSS-induced murine colitis. SIgA but not bLf was identified as a colostral component that altered immune responses and promoted weight gain in colitic mice. Further studies are required to explore how this natural colostral compound modulates the colitis-induced immune cell redistribution.

## Methods

### Mice

Female outbred NMRI mice were purchased from Harlan Winkelmann GmbH (Melderslo, Netherlands) at the age of four to six months. They received standard food and water *ad libitum*. Mice aged eight to twelve months (average body weight 30 g) were randomly assigned to different experimental groups. All animal experiments were performed in compliance with the German animal protection law (TierSchG BGBI S. 1105; 25.05.1998) and were approved by the local commitee on the ethics of animal experiments of the University of Rostock (Landesamt für Landwirtschaft, Lebensmittelsicherheit und Fischerei Mecklenburg-Vorpommern) under permit number 2007/06/14;LALLFM-V/TSD/7221.3-1.1-059/08.

### Colostrum, lactoferrin and secretory immunoglobulin a

For the therapeutic setting, BC powder (SANIMALIS, Heinsberg, Germany) was skimmed, pasteurized, and freeze-dried to ensure minimum denaturation of immunoglobulins and nutrients. The total protein content of the colostrum preparation was 60–70 g/100 g.

The BC (SANIMALIS) solution used for prophylactic treatment was skimmed, casein-free and sterile-filtered. The concentrations of immunoglobulin and other major ingredients were as previously described [5]. The total protein content was 7.25 g/100 ml (carbohydrate 0.2 g/100 ml, fat < 0.01 g/100 ml).

BLf was purified from bovine, and sIgA from human colostrum. IgG was isolated from human serum. All therapeutic agents were purchased from Sigma-Aldrich (Taufkirchen, Germany). Bovine BSA (Sigma-Aldrich) served as control.

### Induction of colitis

Acute colitis was induced by DSS, as previously described [28]. Briefly, DSS (36–50 kDa; MP Biomedicals, Eschwege, Germany) was dissolved in sterilized tap water and presented to the mice at a final concentration of 5% for seven days. Fresh DSS solution was provided every second day.

### Therapeutic treatment protocol

All therapeutic interventions were initiated from day 3 after starting DSS exposure until the end of the experiment (day 15). Either 20 mg/kg bw BC powder (BC, n = 6), 1 mg/kg bw sIgA (sIgA, n = 6), IgG (IgG, n = 6), 150 mg/kg bw bLf (bLf, n = 5) or protein control (BSA, n = 5) were dissolved in physiological sodium chloride solution (0.9% NaCl) and given to each mouse in a final volume of 100 μl daily by oral gavage. DSS-treated negative controls received 100 μl NaCl (DSS, n = 8). Physiological values were obtained from animals with no intervention (untreated, n = 6-8).

### Prophylactic treatment protocol

One hundred μl of pure BC (n = 6) was given to the mice orally by gavage daily for 14 days. All prophylactic treatments were initiated two weeks prior to colitis induction. SIgA and IgG were dissolved in NaCl. Each mouse received 2 mg/kg bw sIgA (n = 8) or IgG (n = 8) in final volume of 100 μl daily for 14 days by oral gavage.

### Clinical evaluation of colitis

After starting DSS exposure at day 1, the mice were weighed daily until the end of the experiment at day 15. Weight loss during the induction phase of colitis was observed from day 1 to day 7 (day 1 = 100%), whereas the recovery phase was defined as weight gain after stopping DSS until the end of the experiment from day 8 to day 14 (day 8 = 100%). Stool consistency was checked for diarrhea and feces were screened for blood during the acute inflammation phase of colitis from day 1 to day 7 (therapeutic protocol) or from day 1 to day 14 (prophylactic protocol) using a HemoCARE occult blood detection kit (Care Diagnostica, Voerde, Germany). DAI was determined according to Cooper et al. (1993) with minor modifications, by scoring changes in fecal blood, and stool consistency, as shown in Additional file 3[29].

### Colon histology

Mice were killed on day 15 after starting DSS. At autopsy, whole colons were removed and their lengths were measured. The colons were unrolled and divided longitudinally into two parts. One part was fixed and embedded in paraffin. Sections were stained with hematoxylin and eosin. The degree of colonic inflammation was analyzed by a pathologist in a blinded fashion, according to the scoring system originally described by Cooper et al. (1993) as shown in Additional file 4[29].

### Flow cytometry

Flow cytometry was performed on leukocytes from peripheral blood, spleens, and mesenteric lymph nodes as described previously [5]. Briefly, leukocytes were labeled using the following fluorescein isothiocyanate (FITC)- and phycoerythrin (PE)-conjugated anti-mouse monoclonal antibodies (mAbs) at the appropriate concentrations: CD3ε FITC, CD19 FITC, *γδ* TCR PE (ImmunoTools, Fiesoythe, Germany), CD11b FITC, CD11c FITC, CD4 PE, CD8 PE; Gr-1 PE (Miltenyi Biotech, Bergisch-Gladbach, Germany), CD83 PE (eBioscience, Frankfurt, Germany). Negative controls consisted of lymphocytes stained with the appropriate isotype controls. Myeloid-derived suppressor cells (MDSC) were defined as CD11b^+^Gr-1^+^. Subsets of these cell populations were detected by gating on the CD11b^+^Gr-1^+high^, CD11b^+^Gr-1^+intermediate^, or CD11b^+^Gr-1^+low^ fraction, definable by varying expression intensity. Samples were analyzed on a FACSCalibur Cytometer (BD Pharmingen) using CellQuest software and gating on total leukocytes (BD Biosciences, Mountain View, CA USA). Relative numbers are given.

### Statistics

Values are reported as the mean ± standard deviation (SD). To ensure reproducibility, experiments were repeated twice with three to four mice per group (except therapeutic experiment group DSS + bLf and DSS + BSA). Data from separate experiments were combined for statistical analysis and presentation in figures and tables. Owing to small sample sizes, group and time effects of clinical parameters (weight loss, DAI) were analyzed by a nonparametric one-way ANOVA model for repeated measurements using SAS 9.2 software [30]. To analyze the impact of DSS-colitis on histological parameters (cell infiltration and distribution, colon length) we first compared between untreated and NaCl groups. To analyze the influence of the treatment during the course of colitis, comparison was made between NaCl and the susceptive treatment group (BC, sIgA, and bLf). For the impact of BC and sIgA, we used BSA as appropriate protein control. To analyze whether observed immunoglobulin-induced effects are specific for sIgA, we used IgG as control. Analyses were performed with the parameter-free Mann Whitney *U*-test using SPSS software [31]. P < 0.05 was considered the criterion for statistical significance.

## Abbreviations

BC: Bovine Colostrum; BSA: Bovine Serum Albumin; bLf: bovine Lactoferrin; CAM: Complementary and Alternative Medicine; DAI: Disease Activity Index; DSS: Dextran Sulfate Sodium; hLf: human Lactoferrin; IBD: Inflammatory Bowel Diseases; IgG: Immunoglobulin G; LPS: Lipopolysaccharide; sIgA: secretory Immunoglobulin A.

## Competing interests

The authors declare that they have no competing interests.

## Authors' contributions

PB and CK designed the research; PB and EZ conducted the research and analyzed the data. PB and SK performed the statistical analyses. PB, CM and CK wrote the paper and have primary responsibility for its final content. All authors read and approved the final manuscript.

## Supplementary Material

Additional file 1: Figure S1 Influence of therapeutics on colon length (A) and histological score (B) of DSS-induced colitis. **Figure S1 **shows the influence of therapeutically applied BC, sIgA, bLf, control protein IgG, and BSA on histological severity of DSS-induced colitis. Colon length (A) and histological score (B) were analyzed at day 15 after starting DSS-exposure as indicated in the methods section. Values are means ± SD (A) or medians as horizontal line (B), of n = 12 (Untreated), n = 8 (NaCl), n = 6 (IgG), n = 5 (sIgA, BC, bLf), and n = 4-5 (BSA) mice. * *P* < 0.01 *vs. *Untreated Mann Whitney *U*-test. Click here for file

Additional file 2: Figure S2 Influence of prophylactic-applied therapeutics on histological severity of DSS-induced colitis. **Figure S2 **shows the histological severity of DSS-induced colitis following prophylactic application of BC, sIgA, and control protein IgG. Values are shown as medians (horizontal line) of n = 6 (sIgA, BC), and n = 5 (IgG) mice. Click here for file

Additional file 3: Table S1 Disease activity index after DSS-exposure. **Table S1 **shows the scoring of the disease activity index after DSS exposure modified according to Cooper HS, Murthy SN, Shah RS, Sedergran DJ: Clinicopathologic study of dextran sulfate sodium experimental murine colitis. *Lab Invest *1993; 69:238–249. The disease activity index is determined by summing the scores for weight loss, occult blood/gross bleeding, and stool consistency (maximal score ;= ;10). Slightly positive stool was defined as incomplete colored occult blood test. Positive occult blood test was defined as complete, intensive colored occult blood test. Gross bleeding was defined as fresh, visible blood on the fur around the anus. Normal stool is defined as well-formed, compact pellets, loose stool is defined as a pasty but formed stool, and diarrhea is defined as liquid stool. Click here for file

Additional file 4: Table S2 Histological scoring of DSS-exposed mouse colons. **Table S2 **shows the histological scoring of DSS-exposed mouse colons. Modified according to Cooper HS, Murthy SN, Shah RS, Sedergran DJ: Clinicopathologic study of dextran sulfate sodium experimental murine colitis. *Lab Invest *1993, 69:238–249. Tissue sections were scored separately for the amount and extent of inflammation, the amount of crypt damage, or regeneration and the percentage of involved tissue. The total histological score was generated by adding the product of the grade and the involvement of each feature to a maximum value of 40. Click here for file

## References

[B1] Triantafillidis JK Merikas E Georgopoulos F Current and emerging drugs for the treatment of inflammatory bowel disease Drug Des Devel Ther 2011 5 185 210 21552489 10.2147/DDDT.S11290 PMC3084301

[B2] Langmead L Rampton DS Review article: complementary and alternative therapies for inflammatory bowel disease Aliment Pharmacol Ther 2006 23 341 349 10.1111/j.1365-2036.2006.02761.x 16422993

[B3] Playford RJ Macdonald CE Johnson WS Colostrum and milk-derived peptide growth factors for the treatment of gastrointestinal disorders Am J Clin Nutr 2000 72 5 14 10871554 10.1093/ajcn/72.1.5

[B4] Solomons NW Modulation of the immune system and the response against pathogens with bovine colostrum concentrates Eur J Clin Nutr 2002 56 Suppl 3 24 28 10.1038/sj.ejcn.1601480 12142957

[B5] Bodammer P Maletzki C Waitz G Emmrich J Prophylactic application of bovine colostrum ameliorates murine colitis via induction of immunoregulatory cells J Nutr 2011 141 1056 1061 10.3945/jn.110.128702 21525246

[B6] Brandtzaeg P Carlsen HS Halstensen TS The B-cell system in inflammatory bowel disease Adv Exp Med Biol 2006 579 149 167 10.1007/0-387-33778-4_10 16620017

[B7] Thoree VC Golby SJ Boursier L Hackett M Dunn-Walters DK Sanderson JD Spencer J Related IgA1 and IgG producing cells in blood and diseased mucosa in ulcerative colitis Gut 2002 51 44 50 10.1136/gut.51.1.44 12077090 PMC1773274

[B8] Murthy AK Dubose CN Banas JA Coalson JJ Arulanandam BP Contribution of polymeric immunoglobulin receptor to regulation of intestinal inflammation in dextran sulfate sodium-induced colitis J Gastroenterol Hepatol 2006 21 1372 1380 16911679 10.1111/j.1440-1746.2006.04312.x

[B9] Carbonare CB Carbonare SB Carneiro-Sampaio MM Secretory immunoglobulin a obtained from pooled human colostrum and milk for oral passive immunization Pediatr Allergy Immunol 2005 16 574 581 10.1111/j.1399-3038.2005.00332.x 16238582

[B10] Sawai T Goldstone N Drongowski RA Coran AG Harmon CM Effect of secretory immunoglobulin a on bacterial translocation in an enterocyte-lymphocyte co-culture model Pediatr Surg Int 2001 17 275 279 10.1007/s003830100593 11409161

[B11] Actor JK Hwang SA Kruzel ML Lactoferrin as a natural immune modulator Curr Pharm Des 2009 15 1956 1973 10.2174/138161209788453202 19519436 PMC2915836

[B12] Buccigrossi V de Marco G Bruzzese E Ombrato L Bracale I Polito G Guarino A Lactoferrin induces concentration-dependent functional modulation of intestinal proliferation and differentiation Pediatr Res 2007 61 410 414 10.1203/pdr.0b013e3180332c8d 17515863

[B13] Debbabi H Dubarry M Rautureau M Tomé D Bovine lactoferrin induces both mucosal and systemic immune response in mice J Dairy Res 1998 65 283 293 10.1017/S0022029997002732 9627847

[B14] Sfeir RM Dubarry M Boyaka PN Rautureau M Tomé D The mode of oral bovine lactoferrin administration influences mucosal and systemic immune responses in mice J Nutr 2004 134 403 409 14747680 10.1093/jn/134.2.403

[B15] Håversen LA Baltzer L Dolphin G Hanson LA Mattsby-Baltzer I Anti-inflammatory activities of human lactoferrin in acute dextran sulphate-induced colitis in mice Scand J Immunol 2003 57 2 10 10.1046/j.1365-3083.2003.01162.x 12542792

[B16] Togawa J Nagase H Tanaka K Inamori M Nakajima A Ueno N Saito T Sekihara H Oral administration of lactoferrin reduces colitis in rats via modulation of the immune system and correction of cytokine imbalance J Gastroenterol Hepatol 2002 17 1291 10.1046/j.1440-1746.2002.02868.x 12423274

[B17] Zhang R Ito S Nishio N Cheng Z Suzuki H Isobe KI Dextran sulphate sodium increases splenic Gr1(+)CD11b(+) cells which accelerate recovery from colitis following intravenous transplantation Clin Exp Immunol 2011 164 417 427 10.1111/j.1365-2249.2011.04374.x 21413942 PMC3087938

[B18] Fischer R Debbabi H Blais A Dubarry M Rautureau M Boyaka PN Tome D Uptake of ingested bovine lactoferrin and its accumulation in adult mouse tissues Int Immunopharmacol 2007 10 1387 1393 17673154 10.1016/j.intimp.2007.05.019

[B19] Warny M Fatimi A Bostwick EF Laine DC Lebel F LaMont JT Pothoulakis C Kelly CP Bovine immunoglobulin concentrate-clostridium difficile retains C difficile toxin neutralising activity after passage through the human stomach and small intestine Gut 1999 44 212 217 10.1136/gut.44.2.212 9895380 PMC1727384

[B20] Hirotani Y Ikeda K Kato R Myotoku M Umeda T Ijiri Y Tanaka K Protective effects of lactoferrin against intestinal mucosal damage induced by lipopolysaccharide in human intestinal caco-2 cells Yakugaku Zasshi 2008 128 1363 1368 10.1248/yakushi.128.1363 18758152

[B21] Tsuchiya T Fukuda S Hamada H Nakamura A Kohama Y Ishikawa H Tsujikawa K Yamamoto H Role of gamma delta T cells in the inflammatory response of experimental colitis mice J Immunol 2003 171 5507 5513 14607957 10.4049/jimmunol.171.10.5507

[B22] Giacomelli R Parzanese I Frieri G Passacantando A Pizzuto F Pimpo T Cipriani P Viscido A Caprilli R Tonietti G Increase of circulating gamma/delta T lymphocytes in the peripheral blood of patients affected by active inflammatory bowel disease Clin Exp Immunol 1994 98 83 88 7923890 10.1111/j.1365-2249.1994.tb06611.x PMC1534185

[B23] Kühl AA Pawlowski NN Grollich K Blessenohl M Westermann J Zeitz M Loddenkemper C Hoffmann JC Human peripheral gammadelta T cells possess regulatory potential Immunology 2009 128 580 588 10.1111/j.1365-2567.2009.03162.x 19807790 PMC2792142

[B24] Boismenu R Havran WL Modulation of epithelial cell growth by intraepithelial gamma delta T cells Science 1994 266 1253 1255 10.1126/science.7973709 7973709

[B25] Komano H Fujiura Y Kawaguchi M Matsumoto S Hashimoto Y Obana S Mombaerts P Tonegawa S Yamamoto H Itohara S Nannot M Ishikawat H Homeostatic regulation of intestinal epithelia by intraepithelial gamma delta T cells Proc Natl Acad Sci 1995 92 6147 6151 10.1073/pnas.92.13.6147 7597094 PMC41659

[B26] Kühl AA Loddenkemper C Westermann J Hoffmann JC Role of gamma delta T cells in inflammatory bowel disease Pathobiology 2002–2003 70 150 155 10.1159/000068147 12571419

[B27] Kühl AA Pawlowski NN Grollich K Loddenkemper C Zeitz M Hoffmann JC Aggravation of intestinal inflammation by depletion/deficiency of gammadelta T cells in different types of IBD animal models J Leukoc Biol 2007 81 168 175 17041003 10.1189/jlb.1105696

[B28] Okayasu I Hatakeyama S Yamada M Ohkusa T Inagaki Y Nakaya R A novel method in the induction of reliable experimental acute and chronic ulcerative colitis in mice Gastroenterology 1990 98 694 702 1688816 10.1016/0016-5085(90)90290-h

[B29] Cooper HS Murthy SN Shah RS Sedergran DJ Clinicopathologic study of dextran sulfate sodium experimental murine colitis Lab Invest 1993 69 238 249 8350599

[B30] Brunner E Domhof S Langer F Nonparametric analysis of longitudinal data in factorial experiments 2002 In Probability and Statistics. New York: John Wiley & Sons

[B31] Mann HB Whitney DR On a test of whether one of Two random variables is stochastically larger than the other Annals Math Stats 1947 18 50 60 10.1214/aoms/1177730491

